# Assessment of cold atmospheric pressure plasma therapy initiated at peak severity in a mouse model of radiation dermatitis

**DOI:** 10.1038/s41598-026-62424-3

**Published:** 2026-07-18

**Authors:** Thoralf Bernhardt, Christin Schlie, Julian Bauer, Karsten Sperlich, Oliver Stachs, Sander Bekeschus, Oliver Friedrich, Brigitte Vollmar, Steffen Emmert, Lars Boeckmann

**Affiliations:** 1 Clinic and Polyclinic for Dermatology, Venereology, and Allergology, Rostock University Medical Center, Rostock, Germany; 2 Rudolf-Zenker-Institute for Experimental Surgery, Rostock University Medical Center, Rostock, Germany; 3 Department of Chemical and Biological Engineering, Institute of Medical Biotechnology (MBT), Friedrich-Alexander-University Erlangen-Nürnberg, Erlangen, Germany; 4 Department of Ophthalmology, Rostock University Medical Center, Rostock, Germany; 5 ZIK Plasmatis, Leibniz Institute for Plasma Science and Technology (INP), Greifswald, Germany

**Keywords:** Gas plasma, Radiodermatitis, Hyperspectral imaging (HSI), Optical coherence tomography (OCT), Multiphoton imaging, Non-invasive diagnostics, Diseases, Health care, Medical research

## Abstract

Radiation dermatitis (RD) is a common side effect of radiation therapy, potentially leading to treatment interruptions. This study evaluated the efficacy of cold atmospheric plasma (CAP) in a mouse model of RD, specifically focusing on treatment initiation at the peak of disease severity. As secondary objective, hyperspectral imaging (HSI) and optical coherence tomography (OCT), were evaluated as objective measures to monitor disease severity. RD was induced via gamma irradiation, with daily CAP treatment initiated at the peak severity. RD severity was monitored using macroscopic scoring and imaging. We found that CAP treatment initiated at the peak of disease did not result in a measurable improved healing under the tested conditions. HSI showed significant differences (*p* ≤ 0.05) between healthy and RD skin even beyond macroscopic scoring. OCT differentiated between healthy and diseased skin, however, no significant difference was observed after macroscopic healing. Our findings suggest that CAP therapy initiated at peak RD does not accelerate recovery. However, this result does not preclude potential efficacy of CAP when administered prophylactically or at the early onset of skin injury. The study underscores the critical importance of treatment timing and highlights HSI and OCT as potentially valuable non-invasive tools for objective RD assessment.

**Supplementary Information:**

The online version contains supplementary material available at 10.1038/s41598-026-62424-3.

## Introduction

Radiation dermatitis is a frequently occurring adverse effect of radiation therapy in cancer treatment^1^. It is accompanied by severe itching and pain and can occur either temporarily during radiation therapy, up to three months afterward, or even persist long-term for more than three months after radiation therapy^2^. Although improvements have been made in the prevention and management of radiation dermatitis, it is still a highly prevalent issue that may require an interruption or even discontinuation of the radiation therapy^3^. Therefore, further improvements for the prevention and treatment as well as for the objective monitoring of radiation dermatitis are needed.

Cold atmospheric pressure plasma (CAP) has emerged as a promising treatment modality for various conditions in dermatology^4^. It has been shown to be an effective therapy to improve wound healing^5,6^ and to reduce pruritus and pain^7,8^. CAP is a partially ionized gas at body temperature consisting of a complex mixture of biologically active components, such as reactive oxygen and nitrogen species (RONS), charged atoms and molecules, electrons, ultraviolet (UV) radiation, visible light, and electromagnetic fields^9^. While there are several publications reporting results from randomized controlled trials showing enhanced healing of chronic wounds in CAP-treated groups compared to control groups receiving standard care^10–13^, so far only two studies have investigated CAP for the treatment of radiation dermatitis^14,15^. In one of the studies, 30 breast cancer patients were treated with a surface discharge DBD (dielectric barrier discharge) plasma device after radiation therapy. Different treatment times and frequencies were tested: 60 s treatment twice a week (3 patients), 180 s three times a week (24 patients), and 120 s five times a week (3 patients)^14^. However, no control group was included. No severe adverse reactions or interactions were reported, and none of the patients indicated the treatment to be unpleasant, whereas all patients would recommend CAP treatment to a friend undergoing radiation therapy for breast cancer. The other study reported a case of a patient with an acute neck radiation dermatitis^15^. Two weeks after radiation therapy the patient experienced severe pain and constant itching and started to treat himself with a needle-type reactor designed for microbiological research and medical applications of CAP. Although this is only a single case without any control, the authors reported accelerated healing, reduced swelling, reduced itching, and abated pain.

While CAP has been extensively studied in preclinical models for wound healing^16–21^ and other dermatological conditions^22,23^, its application in radiation dermatitis, a distinct pathological process with unique challenges, remains underexplored. Specifically, only two prior studies have investigated CAP for radiation dermatitis treatment in humans, both with limitations (e.g., lack of control groups or single-case reports)^14,15^. A study using an X-ray-induced rat radiation dermatitis model showed alleviated radiation-induced skin injury when CAP was applied immediately after first signs of skin lesions appeared^24^. While this work focused on early intervention, the efficacy of CAP treatment beginning at the peak of radiation dermatitis has not been investigated. A major advantage of using animal models is the ability to compare treatment and control groups using individuals with an isogenic background. This reduces confounding factors and increases reliability, reproducibility, and interpretability of results in preclinical studies.

To determine the severity of a radiation dermatitis scoring or grading systems, such as the Radiation Therapy Oncology Group (RTOG) Criteria or the Common Terminology Criteria for Adverse Events (CTCAE), are commonly employed^25,26^. Another skin reaction scoring system comprised of 15 levels (adapted from^27^ was used by Holler and colleagues to score the radiation dermatitis in a mouse model^28^. All these scoring systems evaluate the extent and impact of skin changes caused by radiation therapy. They consider both visual and functional criteria, including erythema (redness), dry or moist desquamation (skin peeling or weeping), ulceration, and associated symptoms such as pain. Since all these criteria are subject to the investigators’ assessment, non-invasive imaging technologies can be considered to objectively monitor the severity of radiation dermatitis. These methods aim at reducing subjectivity by quantifying skin changes, such as redness, swelling, and tissue damage, and providing reproducible data.

Against this background, as a primary objective of the study, we investigated for the first time CAP-mediated healing of radiation-induced dermatitis in mice with treatment initiated at the peak of the disease. As a secondary objective, we assessed hyperspectral imaging and optical coherence tomography as non-invasive objective measures to monitor the severity of radiation dermatitis over time. Label-free multiphoton imaging was used to investigate qualitative differences between healthy and radiation dermatitis tissues.

## Results

**Fig. 1:**
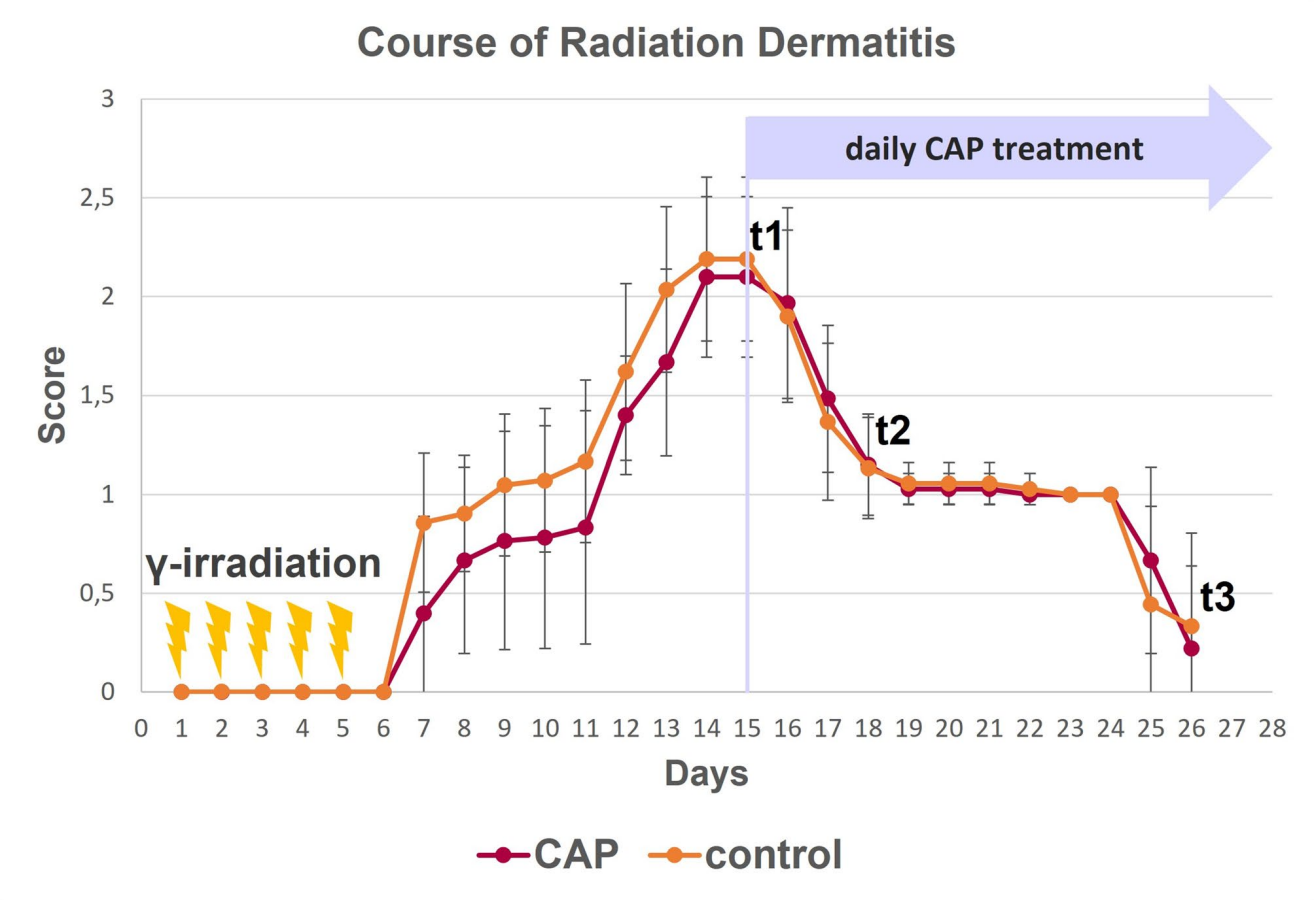
Time course of radiation dermatitis after induction using gamma irradiation. Cold atmospheric pressure plasma (CAP) treatment was started at the maximal score of the disease (t_1_). Values shown are mean scores and standard deviations of *n* = 18 (until t_1_), *n* = 15 (t_1_–t_2_), or *n* = 9 (t_2_–t_3_) mice.

A radiation dermatitis was induced in a mouse model to investigate the efficacy of cold atmospheric pressure plasma (CAP) to enhance healing of radiation dermatitis and to assess hyperspectral imaging (HSI) and optical coherence tomography (OCT) as non-invasive objective measures for the quantification of the severity of radiation dermatitis over time. The severity of the radiation dermatitis was scored over the entire course of the experiment (Fig. 1). First signs of a radiation dermatitis were observed at day seven, two days after the end of the irradiation. The maximal score was reached at days 14 and 15, about 10 days after the end of the irradiation. No statistically significant difference in radiation dermatitis score was observed between untreated and CAP-treated groups. In both groups, the induced radiation dermatitis healed completely within less than two weeks after having reached the maximal severity score.

**Fig. 2:**
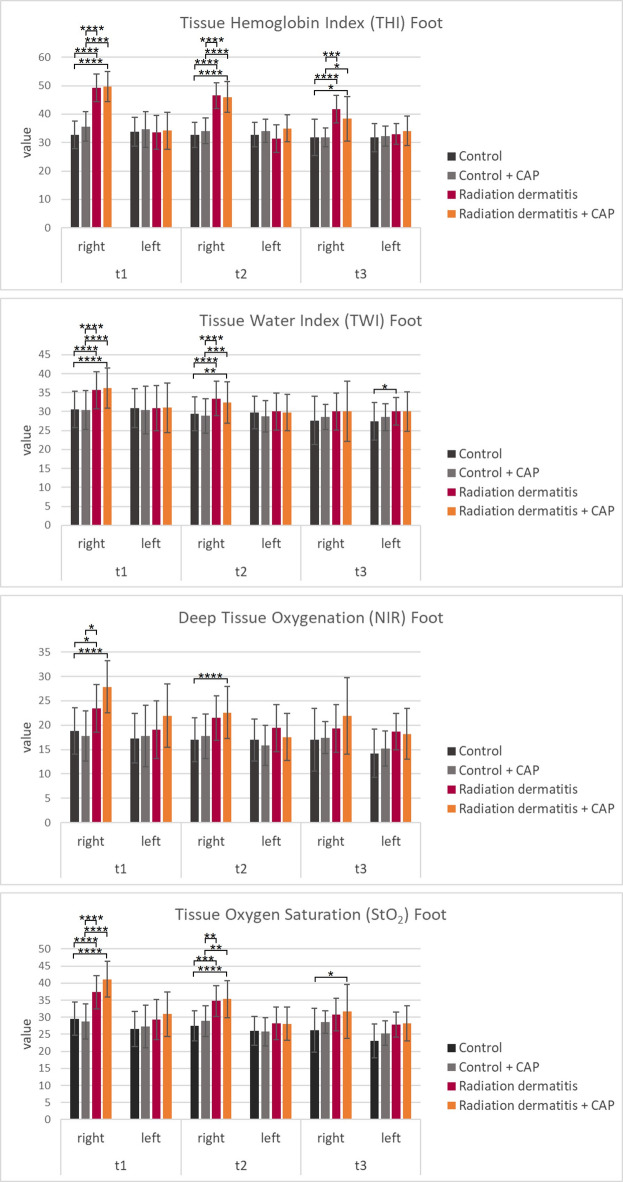
Quantification of different parameters measured by hyperspectral imaging (HSI) at the right (diseased) and left (untreated internal control) hind foot at three different time points (t_1_–t_3_). Values shown are means and standard deviations of *n* = 18 (until t_1_), *n* = 15 (t_1_–t_2_), or *n* = 9 (t_2_–t_3_) mice. Statistical test: Shapiro-Wilk test was performed to confirm normal distribution of data followed by linear mixed-effects model (LMM) with post-hoc comparisons performed using estimated marginal means with Kenward-Roger adjustment for p-values (R packages: lme4, pbkrtest, emmeans). *p < 0.05, **p < 0.01, ***p < 0.001, **** *p* < 0.0001.

Besides the scoring system, HSI and OCT were assessed for their use as objective measures for the severity of the radiation dermatitis. Four different parameters were measured by HSI: tissue hemoglobin index (THI), tissue water index (TWI), deep tissue oxygenation (NIR), and tissue oxygen saturation (StO_2_). Measurements at the feet (right foot was treated and left foot was untreated internal control) revealed a significant difference between untreated control mice and mice with an induced radiation dermatitis at the right foot for all four parameters at time points t_1_ and t_2_ (Fig. 2). Interestingly, even at time point t_3_ when no macroscopic difference was observed anymore, there was still a significant difference in the THI and TWI, indicating that this method can still detect differences between groups when the clinical scoring system shows no difference anymore. As expected, no significant differences between the different groups were observed at any time for the left (untreated) feet. Comparing the four different HSI parameters, differences between healthy mice and mice with radiation dermatitis were most obvious with the THI. Very similar results have been obtained by measurements at the legs, however, with no significant differences at time point t_3_ (Supplementary Figure S1). In line with the result of the macroscopic scoring, neither at the feet nor at the legs a significant difference between untreated or CAP-treated groups was observed. Overall, mice with a radiation dermatitis show increased values for THI, TWI, NIR, and StO_2,_ which decrease over time with declining severity of the disease and hence, HSI and especially the THI provided a good non-invasive and objective measure to monitor the course of a radiation dermatitis.

**Fig. 3:**
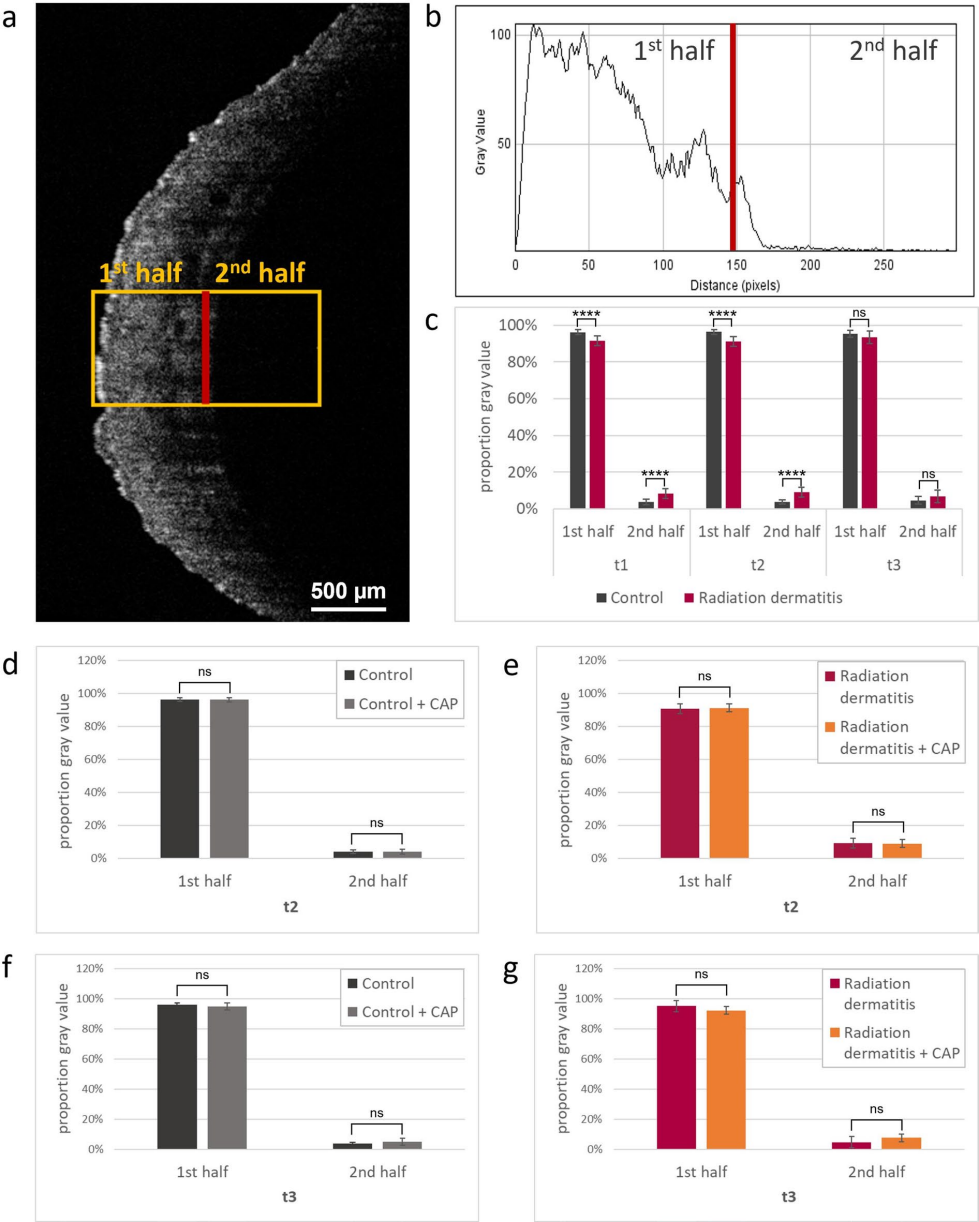
Analysis of images generated by optical coherence tomography (OCT). (**a**) Example image from an OCT measurement at the right hind leg of a SKH1-Hr^hr^ mouse. The orange rectangle indicates the measurement area for subsequent analysis. (**b**) Example of Plot Profile of gray values from left to right within the orange rectangle (**a**). For further analysis, total gray values from the indicated first half (upper layers of skin) and second half (deeper layers of skin) were taken. (**c**) The means of gray values from all images of one group were calculated and compared between different groups (control vs. radiation dermatitis) at three different time points (t_1_–t_3_). (**d**) Comparison of untreated and CAP-treated mice at time point t_2_. (**e**) Comparison of untreated and CAP-treated mice with radiation dermatitis at time point t_2_. (**f**) Comparison of untreated and CAP-treated mice at time point t_3_. (**g**) Comparison of untreated and CAP-treated mice with radiation dermatitis at time point t_3_. (**c**–**g**) Mean values and standard deviations are shown. Statistical test: two-sided t-test. ns = not significant; **** *p* < 0.0001. *n* ≥ 7.

As another non-invasive technology for measuring the severity of the radiation dermatitis, we assessed OCT. Due to the uneven surface of the skin on the feet of the mice, a large variation in the quality between the generated OCT images was observed, which made it difficult to compare the images. Therefore, further analysis was restricted to OCT images of the legs. For these analyses, a defined area was manually determined, ranging from the initial gray values (surface of the epidermis) to deeper layers of the skin, where no OCT signals could be detected (Fig. 3a, orange rectangle [150 × 300 pixels]). For this defined area, a Plot Profile was then created in ImageJ, which represents the distribution of gray values within this area (Fig. 3b).

To identify potential differences between the groups (healthy vs. radiation dermatitis), the mean of gray values in the first half (0 to 150 pixels in depth) and the second half (150 to 300 pixels in depth) was compared. When comparing animals with induced radiation dermatitis to healthy controls, a significant difference was observed at time points t_1_ and t_2_, with an increased proportion of gray values in the deeper layers of the diseased animals compared to the healthy controls (Fig. 3c). This indicates that, in the diseased animals, more light can penetrate into deeper tissue layers before being reflected. Accordingly, a difference in the skin structure due to radiation dermatitis can be measured and quantified using OCT. At the time of macroscopic healing (t_3_), no significant differences in the tissue have been observed using this method.

Next, we analyzed whether there were any differences between the untreated and CAP-treated groups. Confirming the results from scoring and HSI, no differences were found between CAP-treated and untreated healthy mice (Fig. 3d, t_2_ and f, t_3_) nor between CAP-treated and untreated mice with radiation dermatitis (Fig. 3e, t_2_ and g, t_3_).

Taken together, the here employed OCT device with the subsequent analysis of generated gray values in a defined area can show differences between healthy and diseased skin. However, within the constraints of the current analysis approach, it showed lower consistency compared to HSI as an objective, non-invasive measure for monitoring radiation dermatitis.

**Fig. 4:**
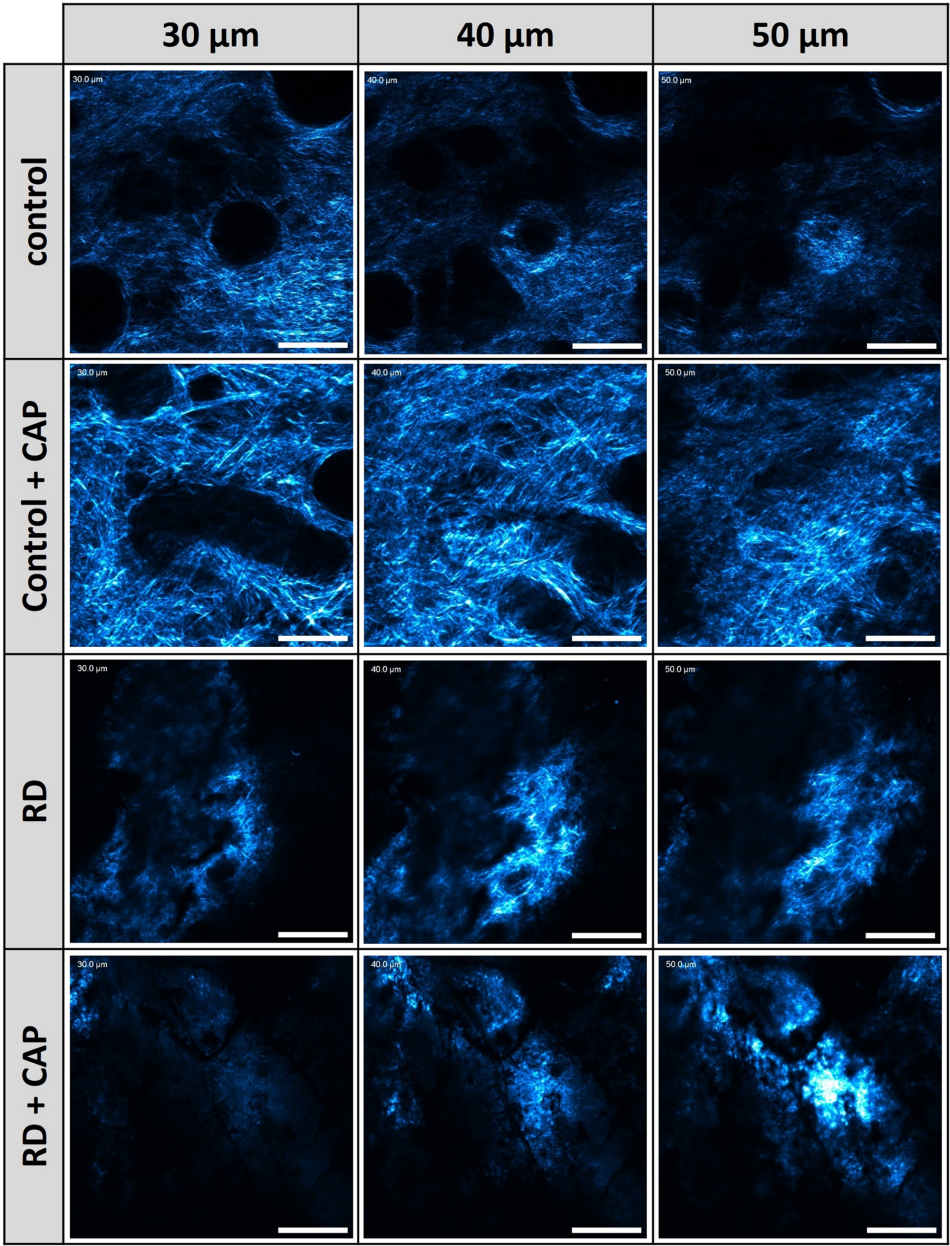
Second Harmonic Generation (SHG) visualization of collagen-I architecture in murine skin samples following induction of radiation dermatitis (RD) and CAP treatment. Panel of label-free SHG images taken at different depths within respective sample groups at time point t_2_. Scale bar: 100 μm.

Finally, the collagen structure in murine tissues was visualized by label-free Second Harmonic Generation (SHG) microscopy. For this, tissues from one mouse per group were qualitatively assessed at time point t_2_. Images of samples from healthy control mice showed a strong SHG signal intensity with collagen-I (the main constituent susceptible to SHG) matrix arranged in the typical isotropic orientation geometry both in the native state and following CAP (Fig. 4). However, a marked disintegration of the isotropic collagen-I network with irregular patterns and signal intensities was observed in tissues of mice with radiation dermatitis, which was also not visibly alleviated by CAP at the given time point.

In summary, the peak of a radiation dermatitis was reached within 10 days after irradiation, and the symptoms disappeared relatively quickly within less than two weeks in both untreated and CAP-treated groups. HSI and especially the THI showed significant differences between healthy skin and radiation dermatitis and, hence, may serve as a sensitive, non-invasive and objective measure to monitor the course of a radiation dermatitis. OCT also revealed significant differences between healthy and diseased skin. SHG microscopy revealed qualitative differences in the collagen structure between healthy and diseased skin.

## Discussion

Our results demonstrate that cold atmospheric pressure plasma (CAP) therapy, when administered at peak disease severity, does not measurably accelerate the recovery process in this murine model. The primary limitation of this study, however, is the timing of CAP intervention, which was initiated at the peak of dermatitis severity. This design specifically evaluates the therapeutic potential of CAP within this narrow window. Furthermore, it is important to note that the radiation dermatitis healed relatively quickly in both untreated and CAP-treated groups. This is in contrast to what other groups have reported for induced radiation dermatitis in mice or rat models. Moriyasu and colleagues induced a radiation dermatitis in C57BL/6 mice using a single 40 Gy dose of a cobalt-60 gamma irradiation source and observed first symptoms about 10 days after irradiation^29^. The symptoms of the radiation dermatitis increased until about day 25 and remained at this level for the rest of the study period until day 60. A single dose of 30 Gy of a cobalt-60 gamma irradiation source was also used in another study to induce a radiation dermatitis at the right hind leg of Sprague-Dawley rats^30^. In that study, animals developed first symptoms three days after irradiation, and the score increased steadily over time with no signs of healing until the last day of the study (day 36). Besides the mouse model, our study also differed in the gamma irradiation source (cesium-137 vs. cobalt-60) as well as in the total irradiation dose, dose rate and treatment scheme (fractionated treatment vs. single dose). A study that also used SKH1-hr mice and fractionated treatment (12 Gy for 4 days = 48 Gy) reported first symptoms about 24 days after treatment^31^. However, in that study, X-rays instead of gamma irradiation were used to induce a radiation dermatitis at the posterior dorsal region of the mice, and no data regarding the further course of the radiation dermatitis were reported. Overall, the radiation dermatitis in our mouse model, even without any treatment, heals fairly quickly compared to others. This rapid spontaneous recovery of the model may have reduced the ability to detect a treatment effect and may explain why no expedited healing could be observed in the CAP-treated group. Furthermore, it is conceivable that starting the CAP treatment at the peak of the disease may be too late. Maybe starting the treatment in parallel to the irradiation or even before irradiation may mitigate the radiation dermatitis. Further studies are required to test this hypothesis. Interestingly, a study performed in rats showed that the severity of a radiation dermatitis can be reduced when CAP treatment is started at first signs of radiation dermatitis^24^. Also limb shortening, which was observed in untreated animals, was prevented by the CAP treatment.

In this study, we have also shown that hyperspectral imaging (HSI) may be used as a sensitive method for quantitative assessment of the severity of radiation dermatitis. Methods that are currently used are primarily based on visual scoring systems such as the Radiation Therapy Oncology Group (RTOG) Criteria or the Common Terminology Criteria for Adverse Events (CTCAE). However, because such assessment is made subjectively, and the results may strongly depend on the researchers’ experience and perceptiveness, several studies have already been carried out to test the suitability of hyperspectral imaging as an objective measure to quantify the severity of a radiation dermatitis or related skin reactions^32^. A study published in 2023 reported on a low-cost algorithms-based hyperspectral imaging (aHSI) system specifically developed for assessment of radiation dermatitis^33^. That system was evaluated on three volunteers and was able to reveal changes associated with skin erythema and could potentially be used to objectively measure the severity of radiation-induced skin reactions. A recently published study proposed a new evaluation method using HSI to improve the accuracy of erythema diagnosis in clinical settings^34^. For that study, 23 subjects diagnosed with atopic dermatitis were investigated, and it was shown that HSI can be applied to erythema severity classification, which can further increase the accuracy and reliability of diagnosis when combined with other features observed in erythema. Therefore, our results align well with previously published results and indicate that HSI is a promising technology for the objective assessment of radiation dermatitis.

As another non-invasive imaging technology for the objective assessment of radiation dermatitis, we investigated optical coherence tomography (OCT) and found that by dividing a defined area in the images into two halves and comparing the gray values of one half to the other (see Fig. 3), differences between groups with healthy skin or radiation dermatitis can be observed. It is important to note that the analytical approach used in this study requires manual selection of a defined region of interest (ROI) and a relatively flat skin surface, so that the rectangle defining the ROI can be placed close to the upper layer of the surface. This represents a limitation for the reproducibility of the analysis. The inconsistency of the images due to uneven surface areas of the skin constitutes another limitation for the evaluation with the device and analytical approach used in this study. Therefore, the results from this analysis should only be interpreted as proof-of-principal rather than as a fully validated monitoring method. A similar but much more sophisticated approach was recently published by Photiou and colleagues^35^. In that study, a traditional feature-based machine learning technique was compared with a deep learning late-fusion approach to classify normal skin versus radiation dermatitis using 1,487 images from 22 head and neck cancer patients. The fully automated algorithm for image segmentation, feature extraction and selection allowed to differentiate healthy skin from radiation dermatitis. Another computational model based on OCT skin scanning to identify and quantify radiation dermatitis was tested in a prospective clinical study including 39 breast cancer patients^36^. This computational model allowed for the identification and quantification of vascularization changes on irradiated skin even in the absence of clinical radiation dermatitis. Overall, our findings together with results from the literature indicate that the OCT technology also shows promise in detecting subtle changes in skin structure and vascularization, potentially allowing for a more accurate and objective measure of radiation-induced skin reactions. Therefore, our results contribute to a promising foundation for future research aimed at developing a quantitative assessment pipeline to enhance the management of radiation dermatitis.

The label-free Second Harmonic Generation (SHG) microscopy revealed qualitative differences in the collagen structure of healthy and diseased skin. However, this data is qualitative and supportive only and does not prove structural tissue alteration.

## Conclusions

This study for the first time explored the effects of cold atmospheric pressure plasma (CAP) treatment of dermatitis in a mouse model. As a secondary objective the utility of non-invasive imaging technologies, such as hyperspectral imaging (HSI) and optical coherence tomography (OCT), in monitoring and assessing radiation dermatitis was investigated. Although CAP treatment initiated at the peak of the disease did not significantly enhance or accelerate the healing of radiation dermatitis under the tested conditions, our findings highlight the rapid natural recovery of this condition in the employed mouse model, suggesting the need for further optimization of experimental parameters, such as treatment timing. This finding does not constitute evidence of CAP’s overall ineffectiveness for radiation dermatitis. Instead, it strongly suggests that the therapeutic window for CAP in this context may be earlier in the disease course, potentially during the initial inflammatory phase or even prophylactically.

Importantly, HSI emerged as a promising and objective tool for evaluating the severity of radiation dermatitis, with tissue hemoglobin index (THI) showing particular promise for monitoring disease progression and resolution. OCT also demonstrated the ability to differentiate between healthy and diseased skin, however, with the device and setting used in this study, the analysis was more challenging due to imaging inconsistencies.

Overall, our results underscore the potential of non-invasive imaging technologies such as HSI as reliable tools for the objective assessment of radiation dermatitis. Further research is expected to unravel the role of CAP in earlier stages after radiation therapy and to refine non-invasive imaging technologies for objective assessment of radiation dermatitis. These advancements could significantly contribute to improving the management and treatment of radiation dermatitis in both preclinical and clinical settings.

## Materials and methods

### Ethics statement

All animal experiments were approved by the local authorities (Landesamt für Landwirtschaft, Lebensmittelsicherheit und Fischerei in Mecklenburg Western Pomerania, approval number: 7221.3-1.1-047/18) and conducted in accordance with the German animal protection law and the EU directive 2010/63/EU.

### Animal experiments

In this work, the mouse strain SKH1-Hr^hr^ (strain code: 477) was used. Animals were bred and provided by the Central Animal Core Facility (Core Facility Zentrale Versuchstierhaltung) of the University Medical Center Rostock. Animals were anesthetized during gamma irradiation as well as during cold atmospheric pressure plasma (CAP) treatment. For the purpose of gamma irradiation and at the time points t1 (max. score), t2 (1/2 max. score) and t3 (score 0), animals were treated with ketamine/xylazine (98 mg/kg ketamine and 6.5 mg/kg xylazine) intraperitoneally to ensure that anesthesia was long enough and sufficient in depth. Because of the absence of an eyelid reflex, an eye ointment (Vidisic Augengel, Dr. Gerhard Mann chem.-pharm. Fabrik, Berlin, Germany) was used to prevent the eyes from drying out. Furthermore, after treatment, the mice were kept warm using a heat lamp until the end of anesthesia. For daily CAP treatment, mice were anesthetized using isoflurane (CP-Pharma, Burgdorf, Germany) and placed on a heating plate maintained at 37 °C to prevent hypothermia during the procedure. After gamma irradiation, the animals were provided with wet food, so they were able to relieve the irradiated leg. The mice were fed *ad libitum* but usually had to climb to get some food, so the wet food was more accessible. Also, potentially occurring pain was alleviated by administering Novaminsulfone through the drinking water (five drops in 100 ml of water). All animals were monitored daily regarding their health condition to prevent unnecessary pain and suffering.

### Induction of radiation dermatitis in a mouse model

The induction of a moderate radiation dermatitis was achieved using a gamma irradiator IBL637 (Gamma Service Medical GmbH, Germany), as described previously^37^. To induce a moderate radiation dermatitis at the right hind leg of the mice, 65 Gy of gamma radiation was applied. Irradiation was performed over five consecutive days using 13 Gy per day.

### Radiation dermatitis scoring

To determine the severity of the induced radiation dermatitis, a scoring metric was used as previously described^37^. According to this metric, a score of 2.5 reflects a moderate radiation dermatitis, which manifests in erythema and moist desquamation in about 50% of the irradiated skin.

### Cold atmospheric pressure plasma treatment

**Fig. 5:**
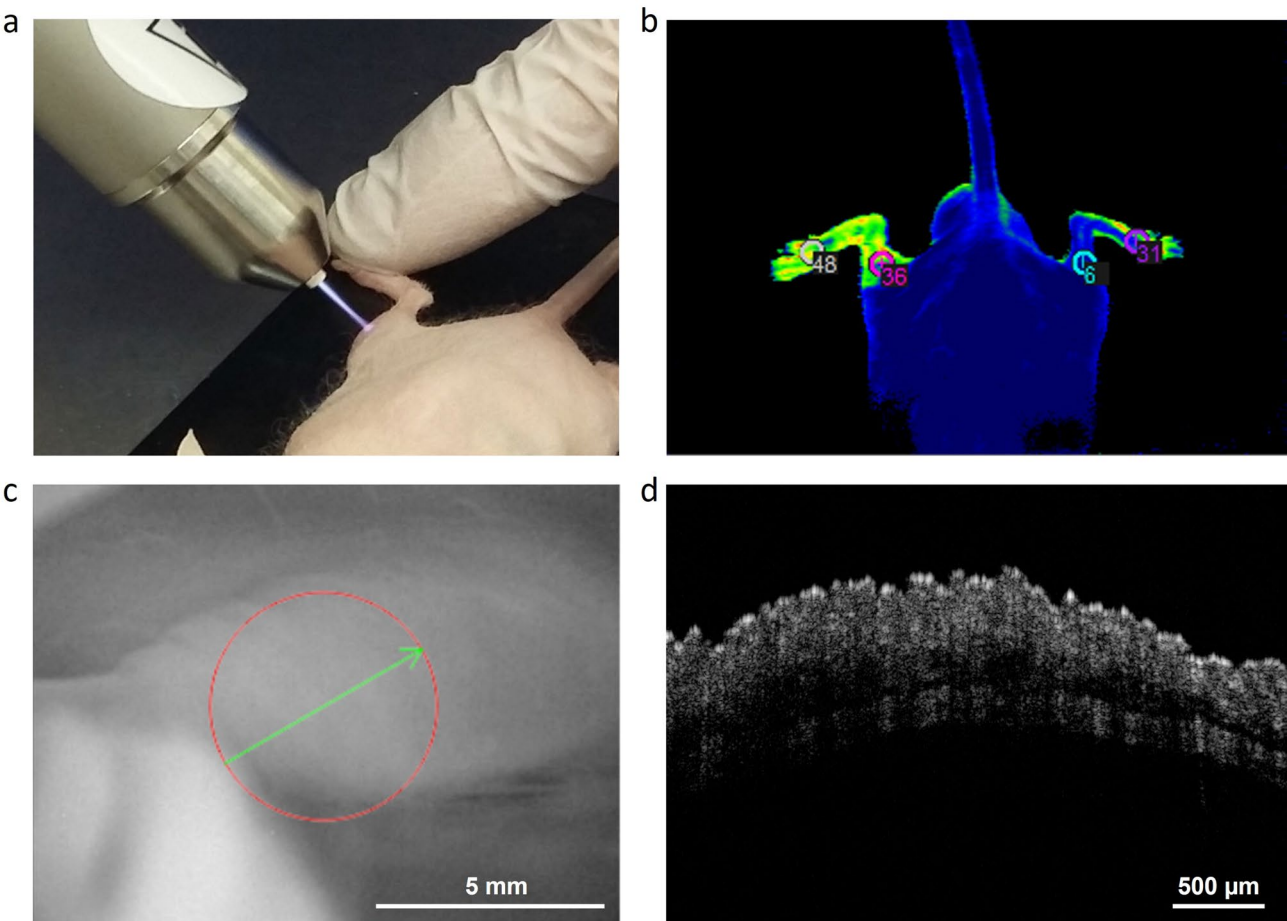
Example images of plasma treatment and imaging. (**a**) Plasma treatment. The right hind leg of SKH1-Hr^hr^ mice was treated daily for 2 min with 4 slm gas flow of argon feed gas. (**b**) Example of false-color representation for the tissue hemoglobin index (THI) of hyperspectral imaging (more example images comparing healthy, diseased, and treated conditions across time points are shown in Figure S2). Circles with numbers show the measurement areas at the feet and legs of the mice. (**c**) Example overview-image of the optical coherence tomography (OCT). The green arrow shows the direction of the scanning. (**d**) The image is an example of an OCT scan along the leg of a mouse.

The treatment with Cold atmospheric pressure plasma (CAP) was performed under anesthesia (2.5 Vol% isoflurane) using the plasma-jet device kINPen MED (Neoplas GmbH, Greifswald, Germany). To reduce variability in the composition of CAP, the device was turned on 10 min prior to its use. The right hind leg of half the irradiated and half of the control mice was treated daily, starting at the time when symptoms of the radiation dermatitis were at peak levels. CAP treatment was performed for 2 min (1 min per cm^2^) with 4 slm gas flow of argon feed gas and a distance of approximately 10 mm between the device and skin (Fig. 5a).

### Hyperspectral imaging

Hyperspectral imaging (HSI) was performed using the TIVITA^®^ Tissue system (Diaspective Vision GmbH, Am Salzhaff-Pepelow, Germany), which captures spectral information in the 500–1.000 nm range with a spectral resolution of 5 nm. HSI enables the recording and visualization of chemical properties with a high spectral resolution, quantifying four parameters of the skin. These parameters are tissue haemoglobin index (THI), tissue water index (TWI), deep tissue oxygenation (NIR) and tissue oxygen saturation (StO_2_). All imaging techniques were performed under anesthesia (ketamine/xylazine). To prevent any influences from other light sources, the room was kept as dark as possible during the HSI recording. To quantify the signal intensity of the different parameters in the images, defined regions of interest (ROI) on the legs and feet of the mice were selected to measure intensity based on the false-color representation (Fig. 5b and Supplementary Figure S2). ROI selection was performed manually by the same investigator at the same anatomical location in an unblinded fashion. The numerical values were subsequently used for the comparison of different groups. Imaging was performed on all animals longitudinally at all three timepoints. However, the group size decreased over time, because some animals were sacrificed at different timepoints to collect skin tissues for later analyses (see study design below). To analyze differences between groups measured by hyperspectral imaging, we used a linear mixed-effects model (LMM) with the lme4 package (v2.0-1) in RStudio (v2026.04.0 + 526). The model included Group (control, control + CAP, radiation dermatitis, radiation dermatitis + CAP), Side (left, right), and Timepoint (t_1_, t_2_, t_3_) as fixed effects, with all possible interactions (Group × Side × Timepoint) to account for potential differential effects across sides and healing stages. Animal-ID was included as a random intercept to account for repeated measurements within the same mouse. Significance of fixed effects was assessed using Kenward-Roger approximation for degrees of freedom (via the pbkrtest package; v0.5.5) to account for the unbalanced design due to sacrifice of mice. Post-hoc pairwise comparisons between groups at t_1_ (baseline, left) were performed using estimated marginal means (EMMs) with the emmeans package (v2.0.3). p-values for these comparisons were adjusted using the Kenward-Roger method to control for multiple testing. The analyses were conducted in R (v4.6.0). Statistical significance was set at *p* < 0.05.

### Optical coherence tomography

Optical Coherence Tomography (OCT) is a non-invasive imaging technique that enables micrometer-scale, cross-sectional visualization of biological tissues based on low-coherence interferometry. In this study, a custom-built swept-source OCT system (IVS-1000, Santec Corporation, Japan) was used to assess morphological skin changes associated with radiation dermatitis in a murine model. The system is equipped with a tunable swept-source laser (HSL-1, Santec Corporation, Japan) operating at a central wavelength of 1,060 nm with a sweep range of 70 nm. The sweep frequency is adjustable between 10 kHz and 400 kHz, allowing for flexible adaptation between imaging speed and sensitivity. At the used settings, the system achieved an axial resolution of approximately 7–10 μm and a lateral resolution of ~ 15 μm in tissue. The typical penetration depth in skin tissue was up to 1.5–2 mm. OCT imaging was performed at defined time points under anesthesia. B-scans of both hind legs and feet were recorded (Fig. 5c and Supplementary Figure S3); however, due to surface irregularities and signal scatter on the plantar skin, quantitative analysis focused on images acquired from the lateral leg region. Image analysis was carried out using ImageJ^38^, where regions of interest (ROI) were manually selected, and gray value distribution profiles were evaluated to quantify structural alterations between healthy and irradiated skin tissue. For each mouse and time point three slices/images (technical replicates) were analyzed. For the analysis a defined area (ROI) of 150 × 300 pixels was manually determined, ranging from the initial gray values (surface of the epidermis) to deeper layers of the skin (an example is shown in Fig. 3a, orange rectangle). The length of the rectangle (ROI) was defined in a way that ensured that all signals from the upper layer of the skin to deeper layers are included (300 pixels). The width (150 pixels) was chosen to be as wide as possible while at the same time allowing for a placement close to the surface without leaving much black space between the boarder of the rectangle and the surface of the signal (epidermis). After defining the ROI this size was used across all images and a Plot Profile for this area was generated in ImageJ (an example is shown in Fig. 3b). The mean of gray values in the first half (0 to 150 pixels in depth) and the second half (150 to 300 pixels in depth) was determined for each image (e.g. 43.07, 46.11, and 44.85 [first half] and 4.15, 5.23, and 4.62 [second half]). Then the mean of the three technical replicates was calculated (e.g. (43.07 + 46.11 + 44.85)/3 = 44.67 [first half] and 4.15 + 5.23 + 4.62 = 4.67 [second half]) and converted into percent values (e.g. 44.67 = 91% [first half] and 4.67 = 9% [second half]). As a result a table with percent values for the first and second half for each mouse was generated. A two-sided t-test was then performed using GraphPad prism 10 to test for significant differences between different groups (control vs. radiation dermatitis) or subgroups (control untreated vs. control CAP-treated and radiation dermatitis untreated vs. radiation dermatitis CAP-treated). Images of about 30% of the mice (28% controls and 32% radiation dermatitis) where the defined area could not be placed in a way that the upper layer of the surface was somewhat parallel to the boarder of the rectangle were excluded. For the comparison of control vs. radiation dermatitis at lease images of 12 mice could be analyzed per group and timepoint. For subgroup analyses images of at least 5 mice per group could be analyzed.

### Multiphoton second-harmonic-generation (SHG) microscopy

Multiphoton imaging was performed using an ultra-fast laser-scanning multiphoton microscope (TrimScope II, LaVision BioTec, Bielefeld, Germany) in combination with a mode-locked femtosecond-pulsed Ti: Sa laser (Chameleon Vision II, Coherent, Santa Clara, CA, USA) at a pulse frequency of 80 MHz and an average input power of 174 mW. To excite collagen SHG-signals, the laser was tuned to an excitation wavelength of 795 nm and focused into the sample through a 25x water immersion objective with a numerical aperture of 0.95 (Leica HC Fluotar L 25x/0.95 W VISIR, Leica Microsystems, Wetzlar, Germany). To assure elliptical light polarization and, therefore, to eliminate polarization-dependent signal intensity variations, a λ/4 wave plate was installed before the objective. The emitted signals were detected in backwards scattered direction with two ultrasensitive photomultiplier tubes (H 7422-40 LV 5 M, Hamamatsu Photonics, Herrsching, Germany), which were equipped with a specific set of dichroic mirrors (495 nm, Chroma ET-series, Chroma Technology Corporation, Bellow falls, VT, USA) and wavelength bandpass filters (405/20 nm for SHG, 525/50 nm for Autofluorescence, Chroma T-series, Chroma Technology Corporation, Bellow falls, VT, USA) for spectral separation. Of each sample, an XYZ volumetric image stack was recorded to allow for three-dimensional morphological collagen fibrillar structure analysis. The imaging parameters were adjusted to a lateral pixel size of 0.4 μm, a line scanning frequency of 1,000 Hz and a step size in axial direction of 0.4 μm. These settings yielded a pixel dwell time of 0.7 µs and a physical cubic voxel size of 0.4 × 0.4 × 0.4 μm. Image analysis and 3D-reconstruction of the image data were done with FIJI/ImageJ^38^.

### Study design

**Fig. 6:**
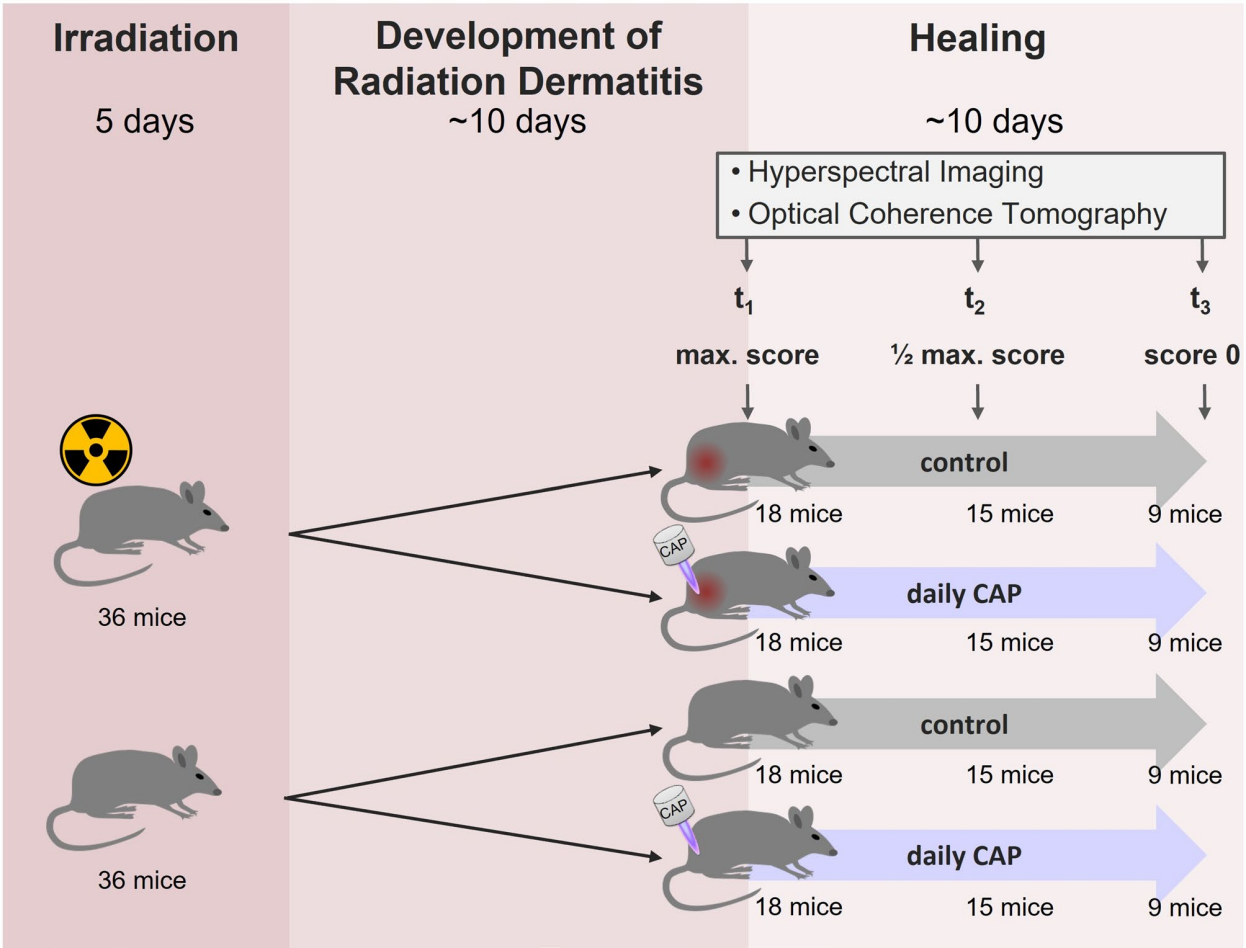
Overview of study design.

A previously established radiation dermatitis mouse model^37^ was used to investigate the efficacy of CAP to enhance healing of radiation dermatitis and to assess HSI and OCT as non-invasive objective measures for the quantification of the severity of radiation dermatitis over time. Therefore, the right hind leg of one group of mice (*n* = 36) was repeatedly exposed to gamma irradiation for five days (Fig. 6). A second group of mice was left untreated as control (*n* = 36). Since not all 72 mice could be handled at once, the mice were divided into six cohorts of 12 mice each. This means per cohort 6 mice were exposed to gamma irradiation and 6 were left untreated. Once the radiation dermatitis in the irradiated mice was fully developed (max. score, t_1_), HSI and OCT were performed in an unblinded fashion and 6 mice form each group (1 mouse per cohort and group) were sacrificed to collect skin samples for later analyses. After HSI and OCT measurements daily CAP treatment of the right hind leg was started for half of the irradiated (*n* = 15 ≙ 2–3 per cohort) and half of the control mice (*n* = 15 ≙ 2–3 per cohort). For randomization mice in each cohort were grouped according to their identification number (lower numbers were CAP treated, higher number served as controls). The identification numbers were assigned by independent animal caretakers before the start of the experiment. Furthermore, approximately the same number of male and female mice were assigned to each group. When the radiation dermatitis score was reduced by half (1/2 max. score, t_2_), and as soon as the dermatitis was macroscopically healed (score 0, t_3_), HSI and OCT were performed again. At t_2_ again 6 mice per group (1 mouse per cohort and group) were sacrificed to collect skin samples. Therefore, the group size for the final timepoint t_3_ was reduced to *n* = 9.

## Supplementary Information

Below is the link to the electronic supplementary material.

Supplementary Material 1

## Data Availability

The data underlying this article are available in the article and in its online supplementary material. Raw data underlying the graphical representations in the article will be shared on reasonable request to the corresponding author.
