# Supplementary material for: Assessment of cold atmospheric pressure plasma therapy initiated at peak severity in a mouse model of radiation dermatitis
Source: Sci Rep. 2026 Jul 18;16:22541. doi: 10.1038/s41598-026-62424-3 (PMC13380627; doi:10.1038/s41598-026-62424-3)
Supplement: Supplementary file 1 — Supplementary Material 1 [file 41598_2026_62424_MOESM1_ESM.docx]

**Supplementary Information**

**
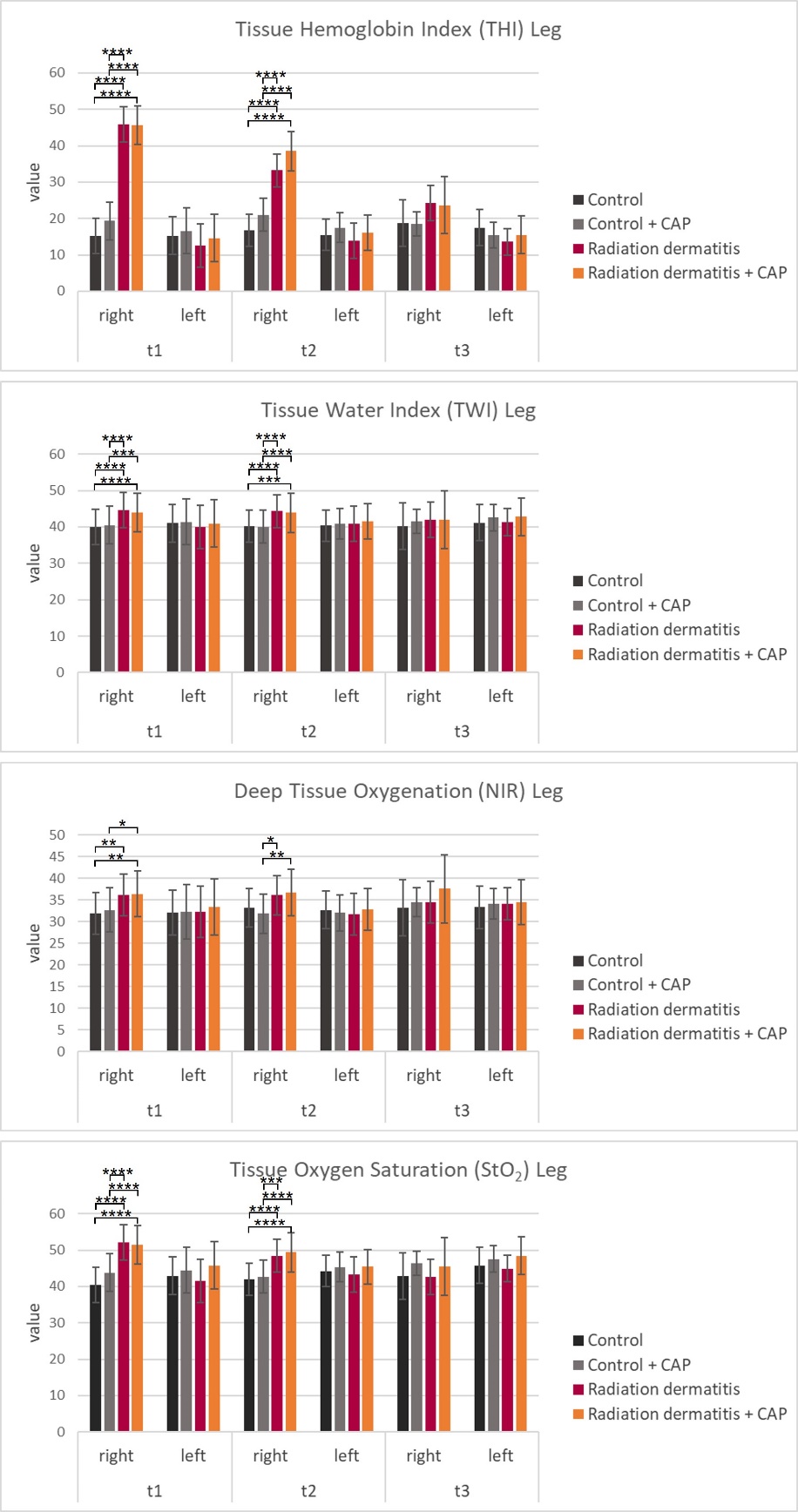
**

**Supplementary Figure S1:** **Quantification of different parameters measured by hyperspectral imaging (HSI) at the right (diseased) and left (untreated internal control) hind leg at three different time points (t_1_-t_3_).** Values shown are means and standard deviations of n=18 (until t_1_), n=15 (t_1_-t_2_), or n=9 (t_2_-t_3_) mice. Statistical test: Shapiro-Wilk test was performed to confirm normal distribution of data followed by linear mixed-effects model (LMM) with post-hoc comparisons performed using estimated marginal means with Kenward-Roger adjustment for p-values (R packages: lme4, pbkrtest, emmeans). *p < 0.05, **p < 0.01, ***p < 0.001, **** p < 0.0001.


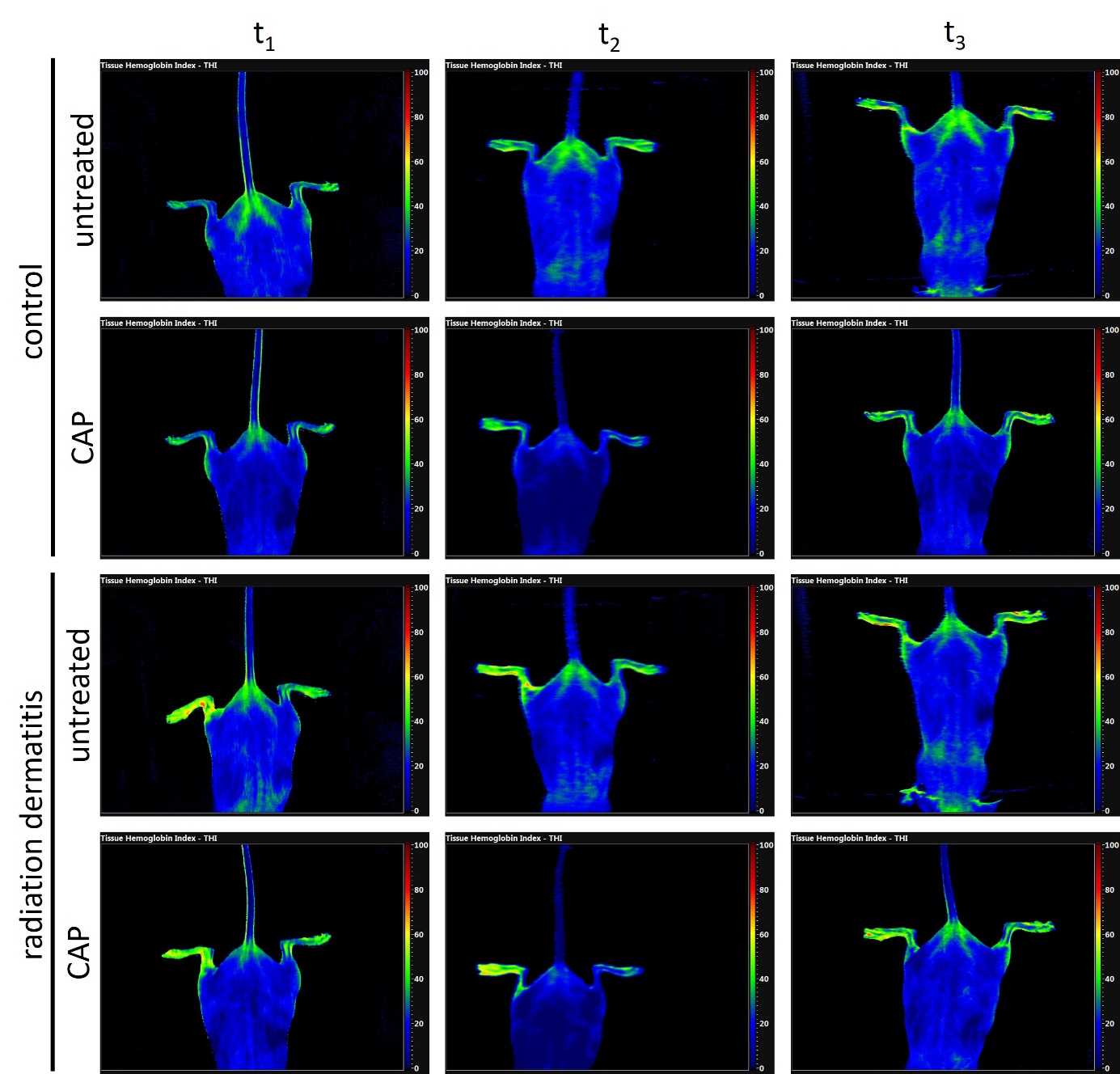


**Supplementary Figure S2:** **Example images comparing healthy, diseased, and treated conditions across time points for the tissue hemoglobin index (THI).**


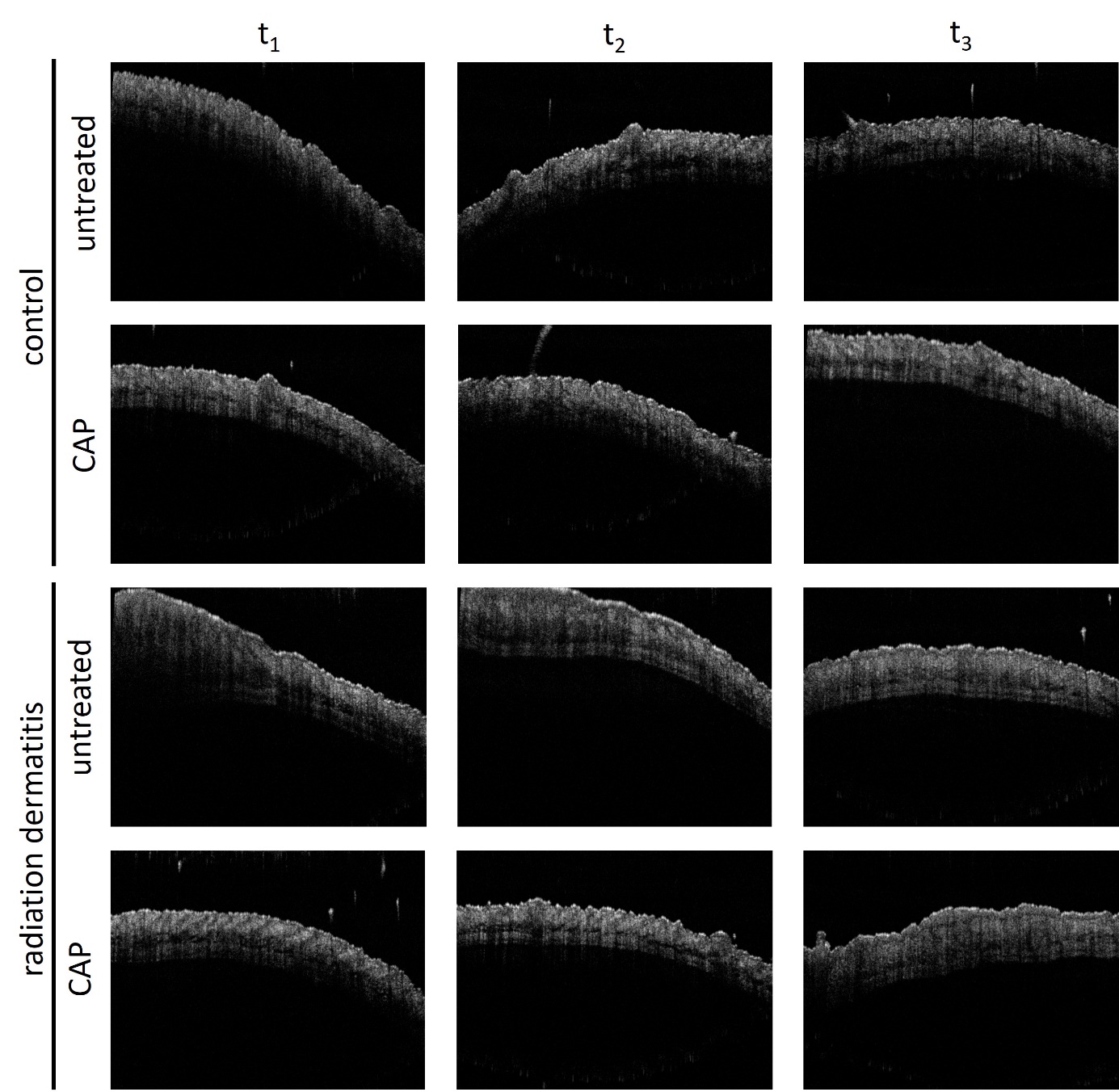


**Supplementary Figure S3:** **Example images comparing healthy, diseased, and treated conditions across time points for OCT.**
